# Twenty-four years of prescription patterns in bipolar disorder inpatients with vs without lithium: a pharmacoepidemiological analysis of 8,707 cases in German-speaking countries

**DOI:** 10.1186/s40345-025-00370-1

**Published:** 2025-02-13

**Authors:** Waldemar Greil, Mateo de Bardeci, Nadja Nievergelt, Andreas Erfurth, Gregor Hasler, Rene Bridler, Sermin Toto, Renate Grohmann, Johanna Seifert, Georgios Schoretsanitis

**Affiliations:** 1 Department of Psychiatry and Psychotherapy, LMU University Hospital, LMU Munich, Zurich, Germany; 2 Psychiatric Private Hospital, Sanatorium Kilchberg, Zurich, Switzerland; 3 Department of Psychiatry, Psychotherapy and Psychosomatics, Hospital of Psychiatry, University of Zurich, Zurich, Switzerland; 4 1st Department of Psychiatry and Psychotherapeutic Medicine, Klinik Hietzing, Vienna, 1130 Austria; 5 Medical University of Vienna, Vienna, Austria; 6 Psychiatry Research Unit, University of Fribourg, Fribourg, Switzerland; 7 Department of Psychiatry, Social Psychiatry and Psychotherapy, Hannover Medical School, Hannover, Germany; 8 The Zucker Hillside Hospital, Psychiatry Research, Northwell Health, Glen Oaks, NY USA; 9 Department of Psychiatry at the Donald and Barbara Zucker School of Medicine at Northwell/Hofstra, Hempstead, NY USA; 10 Psychiatric Department, Ludwig Maximilian University Munich, Nussbaumstr. 7, D-80331 Munich, Germany

**Keywords:** Bipolar disorders, Lithium, Mood stabilizer, Antipsychotics, Antidepressants, Pharmacoepidemiology

## Abstract

**Background:**

Global pharmacoepidemiological evidence suggests dynamically changing prescription patterns in patients with bipolar disorders. We assessed trends in the use of pharmacological agents used in the management of bipolar disorders in inpatients.

**Methods:**

We examined drug use data provided by the Drug Safety in Psychiatry Programme AMSP (German: “Arzneimittelsicherheit in der Psychiatrie”), including psychiatric hospitals in Germany, Austria and Switzerland. We included data from adult inpatients with bipolar disorders (ICD-10: F31) treated between 1994 and 2017. We compared prescription patterns between patients receiving therapeutic regimens with vs. without lithium. Patients with manic and depressive episodes were also analyzed separately.

**Results:**

We identified a total of 8,707 patients (58% females, mean age 50.8 ± 14.8 years). Our analysis revealed a decrease of lithium use (up to 2004) and a consistent increase of prescription rates for second-generation antipsychotics (SGA) among which quetiapine (*n* = 2,677) and olanzapine (*n* = 1,536) were the most common. Among psychotropic drugs, quetiapine was most frequently combined with lithium (*n* = 716, 25.6%). Lithium-treated patients received a higher number of drugs compared to patients not receiving lithium (mean number of drugs in patients with vs. without lithium 4.99, *n* = 2,796 vs. 4.75, *n* = 5,911, *p* = 0.002). Thyroid therapeutics were given more often, valproate and quetiapine less often in the lithium group. Antidepressants were consistently prescribed to more than 60% of patients with bipolar depressive episodes.

**Conclusions:**

Our findings suggest that SGAs are gradually becoming the mainstay treatment option in bipolar disorder, continuously replacing lithium. The use of antidepressants remains concerningly high. We call for action to improve adherence to evidence-based guidelines.

**Supplementary Information:**

The online version contains supplementary material available at 10.1186/s40345-025-00370-1.

## Background

Bipolar disorder is a severe mental illness with a substantial global health burden (Ferrari et al. 2016). The pharmacological treatment constitutes a main part of the short- and long-term treatment of bipolar disorder. Guidelines and experts specifically recommend lithium as a first-line treatment option for bipolar disorder (Yatham et al. 2018; Shah et al. 2017; Fiorillo et al. 2023). In particular, lithium should be also considered in treatment of patients suffering from suicidal ideation, as there is consistent data suggesting suicide preventive properties for lithium (Antolin-Concha et al. 2020; Lewitzka et al. 2015). Moreover, evidence suggests that lithium has long-term immunomodulatory effects (Queissner et al. 2020); specifically, lithium-treatment appears to have anti-inflammatory effects, which in turn are associated with slower progression of illness symptoms (Queissner et al. 2020). Parallel to these disease-modifying properties, evidence from lithium treatment in patients with mild cognitive impairment suggest procognitive effects (Forlenza et al. 2011).

Despite these clinical recommendations and the unique clinical benefits of lithium, prescription trends in patients with bipolar disorder demonstrate that these recommendations are not adhered to (Kriner et al. 2024; Greil et al. 2024). Specifically, a concerning decline in lithium use has been observed in different large pharmacoepidemiological studies (Greil et al. 2024; Rhee et al. 2020; Kessing et al. 2016; Chiu et al. 2024; Shuy et al. 2024; Kleimann et al. 2016; Wolfsperger et al. 2007). Over the past decades, lithium has been gradually replaced by second generation antipsychotics, such as olanzapine and quetiapine, and anticonvulsants, including valproate and lamotrigine, in treatment of patients with bipolar disorder (Kessing et al. 2016; Chiu et al. 2024). However, the alternatives to lithium are associated with serious risks, e.g. olanzapine and quetiapine can cause metabolic syndrome, and valproate should generally be avoided in women of childbearing age (Yatham et al. 2018). To overcome the striking gap between guidelines and clinical practice (Kriner et al. 2024; Kessing 2024), there have been efforts calling for action to counter the declining prescription trends of lithium, e.g. psychoeducation (Malhi et al. 2023), as well as reducing common misconceptions around lithium (Fiorillo et al. 2023). One of the main reasons potentially underlying the prescription trends in patients with bipolar disorder may refer to concerns for adverse lithium-induced reactions in the short- and long-term (Greil et al. 2023; Schoretsanitis et al. 2022; Hidalgo-Mazzei et al. 2023). Additionally, the extensive and elaborate blood monitoring protocols may affect the willingness of patients, but also of clinicians, to initiate lithium over other seemingly low-maintenance treatment options (Hidalgo-Mazzei et al. 2023; Mandal et al. 2019). Such alternative options may include second generation antipsychotics, which are being increasingly prescribed in the treatment of bipolar disorder gradually replacing lithium (Rhee et al. 2020; Chiu et al. 2024). Moreover, the prescription of antidepressant drugs in bipolar patients contrary to recommendations in guidelines reflects a popular treatment choice (Kessing et al. 2016). Nevertheless, pharmacoepidemiological data can help to identify prescription trends. For instance, an Italian population-based study suggested a continuous decline in lithium prescription until 2006, which was subsequently followed by an increase of lithium use in 2010 (Parabiaghi et al. 2015). Similar patterns with increasing lithium prescription rates following early decline were also reported in other large cohorts (Rhee et al. 2020; Shafiq et al. 2023). The incentives underlying such prescription trends are not always easy to elucidate, as they heavily depend on multiple factors, including treatment setting, diagnostic profiles (Shuy et al. 2024), as well as healthcare resources, such as insurance costs and availability of medications and generic drugs (Monteith et al. 2016).

Therefore, it remains essential to provide further prescription data in patients with bipolar disorder. The aim of our study was to investigate drug use patterns in psychiatric inpatients with bipolar disorder over an extensive time period using data from the European drug surveillance programme AMSP (German: “Arzneimittelsicherheit in der Psychiatrie”), additionally comparing the use of drugs in patients suffering from bipolar disorder with and without lithium. Such evidence will not only facilitate the identification of gaps between clinical practice and evidence-based recommendations, but could also provide a foundation to determine if future interventions are necessary.

## Methods

The present study analyzed drug use data from the European Drug Safety in Psychiatry (AMSP) Programme (Bridler et al. 2015; Glocker et al. 2023; Seifert et al. 2022), an ongoing multicenter drug safety project in German speaking countries. AMSP gathers data on the use of psychotropic drugs as well as related reports of adverse drug-induced reactions (ADRs) from a trinational network of psychiatric hospitals in Austria, Germany and Switzerland. Collected data includes drug use, age, sex, as well as diagnoses of all inpatients in treatment on two reference days per year in a cross-sectional manner. A detailed description of methods can be found elsewhere (Bridler et al. 2015; Glocker et al. 2023; Seifert et al. 2022; Grohmann et al. 2004).

### Statistics

We classified the prescribed drugs into the following groups antidepressant drugs (AD), antipsychotic drugs (APD), tranquilizing drugs (TRD), hypnotic drugs (HYP), lithium (Li) and anticonvulsant drugs (AC). We included patients between ≥ 18 and < 90 years of age with a primary diagnosis of bipolar disorder (F31 according to International Statistical Classification of Diseases and Related Health Problems, 10th Revision; ICD-10) between 1994 and 2017 (up to 2000 patients were diagnosed by the clinicians according to ICD-9, but classified according to ICD-10 for this study). Patients were allocated to each group based on at least one prescription of a drug from each group for every calendar year from 1994 to 2017. Finally, we calculated the percentages and visualized the results. Given that mood stabilizers are the mainstay of treatment for bipolar disorder (Schoretsanitis and Paulzen 2022), we specifically assessed the prescription patterns of mood stabilizers separately: we investigated the use of second-generation antipsychotics (SGA), mood stabilizers (MS; including lithium and the anticonvulsants carbamazepine, lamotrigine and valproate), lithium, as well as AC (all anticonvulsants) separately.

### Combinations with vs. without lithium in patients with bipolar disorder

Further, we aimed to address drug use patterns in patients with vs. without lithium. We also calculated the percentage of psychotropic drugs in patients with vs. without lithium. P-values were determined using chi-square methods.

### Diagnostic subtypes

Drug use patterns in patients with depressive episodes (F31.3-5) and manic episodes (ICD-10 F31.0-2) were examined separately.

## Results

**Fig. 1:**
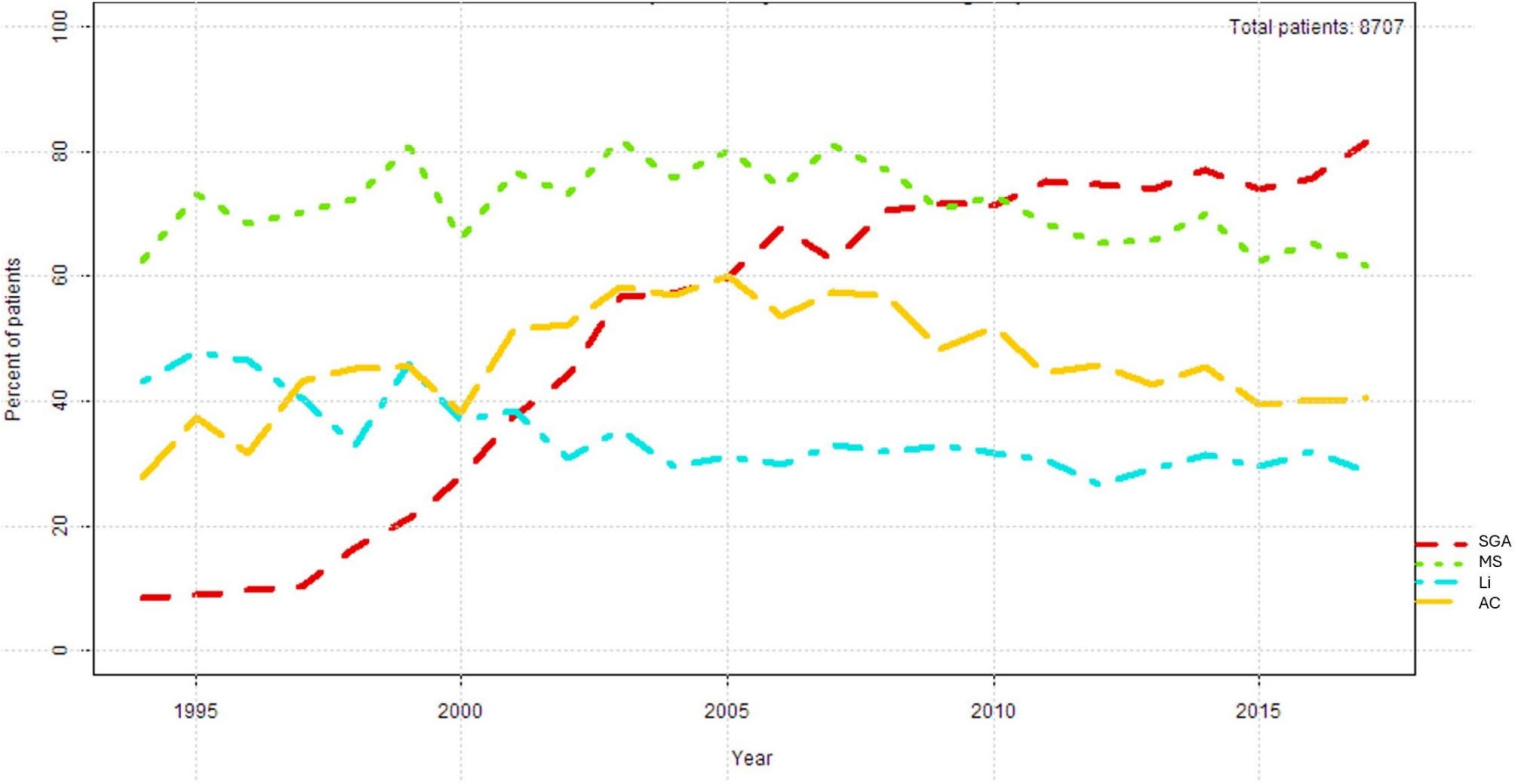
Use of second-generation antipsychotics (SGA), of lithium, of anticonvulsants (AC), and of mood stabilizers, in patients with bipolar disorders from 1994 to 2017. MS: Mood Stabilizers (lithium, carbamazepine, lamotrigine, valproate). SGA: Second Generation Antipsychotics. Li: Lithium. AC: Anticonvulsant medication

We investigated drug use patterns in 8,707 adult patients (58% females, mean age 50.8 ± 14.8 years, Table 1). The temporal course of prescriptions of SGAs and mood stabilizers (i.e., lithium, valproic acid, lamotrigine, and carbamazepine), lithium (separately) and anticonvulsants between 1994 and 2017 is shown in Fig. 1. The exact number of prescriptions can be found in supplementary Table 1. We found an increase of prescription rates for SGAs (up to about 80%), most commonly quetiapine (*n* = 2,677) and olanzapine (= 1,536; supplementary Table 2). Quetiapine was most frequently combined with lithium (*n* = 716, 25.6%). Quetiapine was used in low doses as follows (up to 25 mg/d: 3.8%, up to 50 mg/d: 11%, up to 75 mg/d: 13% and up to 100 mg/d: 23%), and olanzapine as follows (up 2.5 mg/d: 3.7%, up to 5 mg/d: 17.5% and up to 7.5 mg/d: 22.3%). Lithium use declined from about 45% to about 30% in the study period (stable at this level from 2004 on), while the mood stabilizers (i.e., lithium plus mood stabilizing anticonvulsants) remained largely stable with about 60% throughout the whole study period. Valproic acid was used more frequently in men than in women (approximately 40% versus approximately 30%). Notably, valproate was also used in 20–30% of cases in women aged 40 years and younger, compared with about 40% in men aged *≤* 40 years (see supplementary Fig. S1 a, b, c, d).

### Therapeutic regimens with vs. without lithium in patients with bipolar disorder

**Fig. 2:**
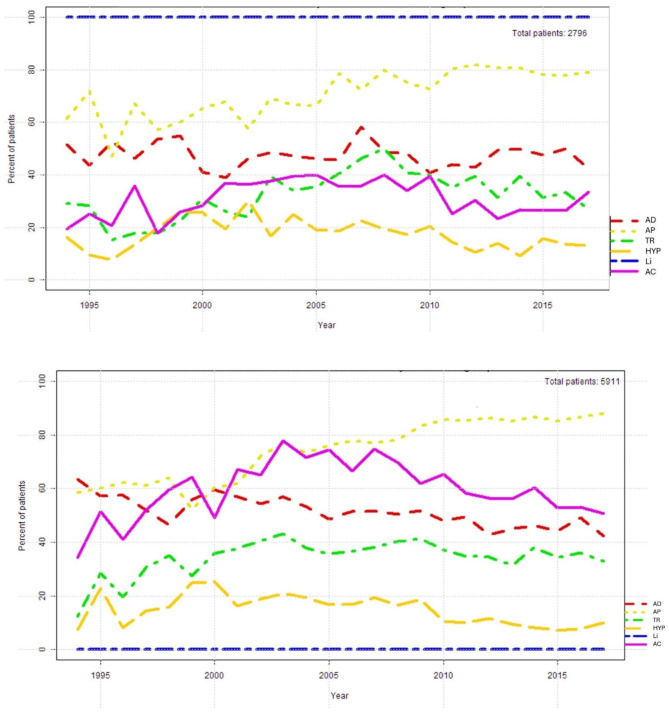
Use of psychotropic drugs in patients in bipolar disorders treated with lithium (a, above) and in patients treated without lithium (b, below). The exact values per year for the figure can be found in the supplement material (supplementary tables). AD: Antidepressant drugs. AP: Antipsychotic drugs. TR: Tranquilizers. HYP: Hypnotics. Li: Lithium. AC: Anticonvulsant drugs

The temporal course of prescription rates for antipsychotics, anticonvulsants, antidepressants, hypnotics and tranquilizers in patients with (*n* = 2,796; mean age 49 + 14 years) vs. without lithium (*n* = 5,911; mean age 50 *±* 15 years) is shown in Fig. 2a and b (See Table 2).

In the without-lithium group anticonvulsants were used more often, whereas antipsychotics did not differ essentially. A list of the most common drugs combined with lithium is provided in Table 2a, b and in supplementary table 3a, b. Among psychotropic drugs, valproate was more commonly used in the lithium-free group (38% vs. 16%, *p* < 0.001), as was quetiapine, but to a lesser extent (33% vs. 26%, *p* < 0.001). The same applies to lorazepam, lamotrigine, carbamazepine and aripiprazole. Note, in the period 2008–2017 quetiapine use was very high in both groups, without lithium as well as with lithium (47% vs. 38%, *p* < 0.001; supplementary table 3b).

**Table 1:** Patients with bipolar disorders with data on prescription patterns between 1994–2017 ICD-10: International Statistical classification of diseases and related health problems 10th revision; SD: standard deviation

Total number of patients	8,707
Age, years (SD)	50.8 (14.8)
Females, n (%)	5,073 (58%)
> 65 years old, n (%)	1,543 (18%)
Diagnostic subgroups (ICD-10)	
Depressive episodes (F31.3-5)	4,023 (46.2)
Manic episodes (F31.0-2)	3,305 (38.0)
Other (F31.6-9)	1,194 (13.7)
No information on subdiagnoses (F31.x)	185 (2.1)

**Table 2:** Rates (%) of the most common psychotropic (a) and somatic medications (b) in bipolar disorders without (*n* = 5911) and with lithium (*n* = 2796), 1994–2017

(**a**)				
Psychotropic medications	Without Lithium		With Lithium	
	*n*	%	*n*	%
Valproaic acid*	2261	38.25	449	16.06
Quetiapine*	1961	33.18	716	25.61
Lorazepam *	1270	21.49	513	18.35
Olanzapine	1058	17.90	478	17.10
Lamotrigine*	855	14.46	225	8.05
Venlafaxine	616	10.42	287	10.26
Mirtazapine	609	10.30	271	9.69
Risperidone	592	10.02	240	8.58
Diazepam	568	9.61	300	10.73
Carbamazepine*	531	8.98	133	4.76
Aripiprazole *	501	8.48	144	5.15

(**b**)				
Somatic medications	Without Lithium		With Lithium	
	*n*	%	*n*	%
Antihypertensive agents*	2081	35.20	763	27.30
Gastrointestinal agents*	1127	19.07	398	14.23
Thyroid therapeutics*	946	16.10	593	21.21
Analgetics/antirheumatics*	907	15.34	303	10.84
Diuretic agents*	628	10.62	188	6.72
Antidiabetic agents	550	9.30	215	7.69
Lipid-lowering agents	450	7.61	193	6.9
Vitamins	335	5.67	148	5.29
Laxatives	273	4.62	158	5.65
Anticoagulant drugs*	242	4.09	46	1.65

* *p* < 0.001

Among somatic medications, thyroid therapeutics were used more often in the lithium vs. without-lithium group (21% vs. 16%, *p* < 0.001, Table 2b). Other groups of somatic medications were given more frequently in the lithium-free versus lithium-treated bipolar patients. Note, antihypertensive agents including beta-blockers, calcium antagonists and angiotensin-converting enzyme (ACE) inhibitors and other drugs for the treatment of hypertension, were most frequently combined with lithium (*n* = 763, 27%). However, these drugs were even more used in the without-lithium group (*n* = 2081, 35%, *p* < 0.001). Furthermore, statistically significantly more often given in the lithium-free group of patients were gastrointestinal agents, analgetics/ antirheumatics, and diuretic agents (Table 2b).

Patients under lithium were treated with a higher number of drugs (both psychotropic and somatic) compared to those not treated with lithium (mean number of drugs in patients with vs. without lithium 4.99, *n* = 2796 vs. 4.75, *n* = 5911, *p* < 0.01), supplementary Table 4). When considering only use of psychotropic drugs, lithium-treated patients received a higher number of psychotropic drugs (including lithium) compared to patients not treated with lithium (mean number of drugs in patients with vs. without lithium (3.4 vs. 2.9, *p* < 0.01). Daily doses of concomitantly used drugs did not differ between patients with vs. without lithium except for lamotrigine, valproate and venlafaxine (with higher median doses in the lithium group; supplementary Table 5). The main difference between lithium and non-lithium treatment is the higher use of anticonvulsant medication (AC).

### Prescriptions in different diagnostic groups of patients with bipolar disorder

#### Patients with depressive episodes (ICD-10 F31.3-5)

**Fig. 3:**
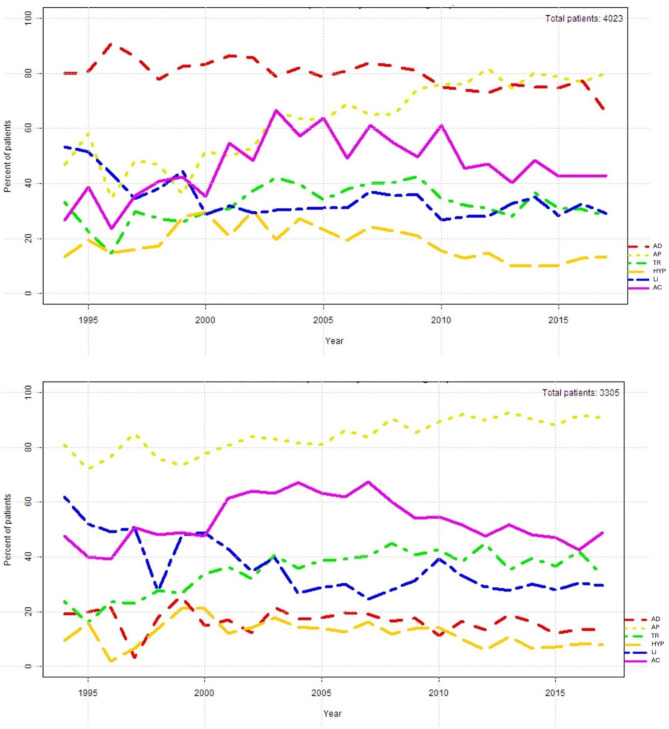
Use of psychotropic medications in 4,023 patients with depressive episodes (ICD-10 F31.3-5; a, above) and in 3,305 patients with manic episodes (ICD-10 F31.0-2; b, below). The exact values per year for the figure can be found in the supplement material (supplementary Tables 6 and 7). AD: Antidepressant drugs. AP: Antipsychotic drugs. TR: Tranquilizers. HYP: Hypnotics. Li: Lithium. AC: Anticonvulsant drugs

The temporal course of prescriptions for lithium, anticonvulsants, antidepressants, antipsychotics, tranquilizers and hypnotics in 4,023 patients (mean age 52.7 ± 14.4 years, 20% are > 65 years old, 62% are females) during a depressive episode shown in Fig. 3a, whereas the yearly prescription rates can be found in supplementary Table 6. More than 65% (up to 90%) of patients with bipolar depressive episodes were treated with antidepressants throughout the study period. Lithium use declined from about 50% to about 30% (until 2000) and remained more or less stable in the following years.

### Patients with manic episodes (ICD-10 F31.0-2)

The temporal course of prescriptions for lithium, anticonvulsants, antidepressants, antipsychotics, tranquilizers and hypnotics in 3,305 patients (mean age 49.0 ± 15.2 years, 15% are > 65 years old, 53% are females) with manic episodes during the study period is shown in Fig. 3b, whereas the yearly prescription rates can be found in supplementary Table 7. Remarkedly, the use of lithium declined clearly from 62 to 29% (no essential change from 2004 on). The antipsychotic drugs remained stable at about 80% throughout the study period (see also supplementary Table 7; supplementary Tables 8 and 9 give the exact rates of drugs in all bipolar patients with and without lithium). The comparison of manic patients without and with psychotic symptoms (F 31.1 versus F 31.2) shows an increase in antipsychotic use from about 70% to over 80% in mania without psychosis, while in psychotic mania antipsychotic use was about 80% over the whole period (supplementary Fig. S2 a, b).

## Discussion

Our analysis of a large-scale pharmacoepidemiological dataset aimed to capture drug use patterns in inpatients with bipolar disorder in a network of hospitals in German-speaking countries over 24 years. Our focus here was also on drug use in patients with and without lithium. Our evidence aligns with trends from previous studies in patients with bipolar disorder from the USA, Taiwan, Denmark and other countries, reporting an essential increase of antipsychotics over the past years (Rhee et al. 2020; Kessing et al. 2016; Chiu et al. 2024). This increase was primarily driven by the consistently increasing prescription of SGAs, such as quetiapine, as observed in other cohorts (Rhee et al. 2020), whereas the use of first-generation antipsychotics gradually declined (Rhee et al. 2020; Chiu et al. 2024). We also found that the combined use of SGAs and lithium increased over the years in our cohort. It should be emphasized that SGAs are given at low doses such as quetiapine up to 100 mg/d and olanzapine up to 7.5 mg/d in about 20% of cases. Furthermore, antipsychotics were also administered in a high percentage of manic patients without psychotic symptoms. It has been previously suggested that the intensive marketing following the approval of SGAs may account for the substantial increase of prescriptions of SGAs in patients with bipolar disorder (Rhee et al. 2020). Initially prescribed off-label, SGAs were gradually approved for the treatment of bipolar disorder after demonstrating efficacy in bipolar depression. Because bipolar depression is considered difficult to treat, the efficacy of SGAs in this indication may have further contributed to the increased use of SGAs in bipolar disorder (Rhee et al. 2020). Simultaneously, misconceptions regarding lithium’s safety and the required blood monitoring protocols may have also led clinicians to favor SGAs over lithium (Fiorillo et al. 2023; Rhee et al. 2020).

In fact, in our cohort we observed a gradual decline of lithium use, which was relatively more pronounced in patients with manic episodes. The decline of lithium prescriptions has been also reported in other countries (Rhee et al. 2020; Kessing et al. 2016; Lyall et al. 2019; Lin et al. 2022). Regardless of the reasons behind the replacement of lithium with SGAs in the treatment bipolar disorder, it remains difficult to predict whether the declining use of lithium will eventually subside (Rhee et al. 2020; Parabiaghi et al. 2015; Shafiq et al. 2023). However, as clinicians gain more experience with SGAs in treatment of bipolar disorder (Koistinaho et al. 2023), it is unlikely that this trend will change in the near future. That the decline in lithium use ended after 2004 in our study is consistent with the findings of Kriner et al. 2024 (Kriner et al. 2024), who examined lithium use after 2014.

In our cohort, lithium-treated patients were, on average, treated with more drugs than patients without lithium. Polypharmacy is a common pattern in the treatment of patients with bipolar disorder (Rhee et al. 2020; Koistinaho et al. 2023). Moreover, patients treated with lithium may comprise a patient subgroup with a more complex illness course less responsive to pharmacotherapy; consequently, polypharmacy is a common treatment approach involving not only psychotropic, but also somatic drugs. For instance, the use of lithium in combination with other psychotropic drugs may suggest reluctance of clinicians to prescribe lithium unless all other options have been exhausted. The high frequency of use of antihypertensive drugs in combination with lithium and even more so in patients without lithium may be related to high rates of comorbidity and regular somatic monitoring in drug-treated bipolar patients (Nederlof et al. 2018). There may be several reasons for the high frequency of the combination with thyroid drugs; changes in thyroid function may be observed in patients with first-episode bipolar disorder, even in those not treated with lithium or quetiapine (Song et al. 2023). Moreover, thyroid hormone augmentation is used in treatment with bipolar disorder, in particular in treatment resistant depression, also in the absence of thyroid disease (Seshadri et al. 2022). Addition of thyroid hormones is common in lithium treatment to counteract latent hypothyroidism. Also, the risk of clinically relevant hypothyroidism in patients receiving long-term treatment with lithium has been previous reported (McKnight et al. 2012).

Another remarkable finding refers to the high rates of prescriptions of antidepressants, contrary to recommendations in guidelines, particularly in patients with bipolar depressive episodes, although a small decline was observed over the years. Clinicians should be aware of the risks related to antidepressant use in patients with bipolar disorder, such as manic switch (Jefsen et al. 2023). In this context, the use of antidepressants in patients without lithium was also worryingly high (between 66% and 91%), where lithium may have been co-prescribed to prevent a manic switch. Previous analyses of AMSP data have also observed a decrease in lithium and increase in valproate use in manic episodes (Kleimann et al. 2016; Wolfsperger et al. 2007), high antidepressants use in bipolar depression (Greil et al. 2012; Haeberle et al. 2012), and polypharmacy in both poles of bipolar disorder (Kleimann et al. 2016; Wolfsperger et al. 2007; Greil et al. 2012; Haeberle et al. 2012). Using valproic acid in women aged 40 and younger was also acknowledged and criticized in an earlier AMSP paper (Kleimann et al. 2016).

### Limitations

Several limitations should be considered when interpreting our findings. First, the lack of data concerning treatment outcomes, such as clinical response and/or safety does not allow any conclusions on effectiveness and tolerability of the prescribed drugs. Second, our pharmacoepidemiological dataset only contains prescription data for inpatients; however, previous evidence suggests that the treatment setting, i.e., out- vs. inpatient (Al Jurdi et al. 2012), may have a relevant impact on prescription patterns given the major difference of clinical profiles of patients and thus we suggest caution when it comes to extrapolating our findings to the outpatient treatment. In fact, we may expect that use of lithium in the outpatient setting may be even more spectacularly declining than the trend we reported in inpatients in our study. Third, diagnoses were extracted from the medical records and therefore they may be less reliable compared to structured interviews. Finally, the dataset analyzed does not include specific clinical details of the cases, such as illness duration, hospitalization frequency or duration, suicidal tendencies, or family history. In addition to the large size of the data set, a further strength of the study is that prescriptions correspond to the actual use of the drugs, as the data is based on inpatients.

## Conclusions

In this study, we assessed pharmacoepidemiolocal trends on the treatment of inpatients with bipolar disorder in a network of psychiatric hospitals in three European countries over a period of almost a quarter of century. We detected a consistently increasing use of SGAs, progressively replacing lithium. On the other hand, lithium appears to be frequently embedded in polypharmacy, suggesting that it is used in less responsive patients. The vanishing use of lithium in favor of SGAs and the persistently high rates of not recommended prescriptions of antidepressants have been previously reported in other parts of the world and call for action to reduce the gap between evidence-based recommendations and clinical practice.

## Electronic supplementary material

Below is the link to the electronic supplementary material.

Supplementary Material 1

## Data Availability

No datasets were generated or analysed during the current study.
