# Supplementary material for: Twenty-four years of prescription patterns in bipolar disorder inpatients with vs without lithium: a pharmacoepidemiological analysis of 8,707 cases in German-speaking countries
Source: Int J Bipolar Disord. 2025 Feb 13;13:3. doi: 10.1186/s40345-025-00370-1 (PMC11825962; doi:10.1186/s40345-025-00370-1)

**Supplementary material**

**Twenty-Four Years of Prescription Patterns in Bipolar Disorder Inpatients with vs without Lithium: A Pharmacoepidemiological Analysis of 8,707 Cases in German-Speaking Countries**

Waldemar Greil, Mateo de Bardeci, Nadja Nievergelt, Andreas Erfurth, Gregor Hasle5, René Bridler, Sermin Toto, Renate Grohmann, Johanna Seifert, Georgios Schoretsanitis

Contents

[Supplementary Table 1. Numbers (%) of prescriptions of second-generation antipsychotics (SGA), mood stabilizers (MS) in total, lithium (Li) and anticonvulsants (AC) for each study year 2](#_Toc188425867)

[Supplementary Table 2. Rates of second-generation antipsychotics (SGA) prescribed; SGAs with less than 25 prescriptions are not shown. 3](#_Toc188425868)

[Supplement Table 3: Rates (%) of the 10 most common psychotropic drugs in bipolar disorders without and with lithium (a) 1998-2007 (b) 2008-2017 4](#_Toc188425869)

[Supplementary Table 4. Mean number and standard errors (SE) of medications in bipolar patients treated with (n = 2796) vs. without lithium (n = 5911) per year, including psychotropic and non-psychotropic medications. 5](#_Toc188425870)

[Supplementary Table 5. Median daily doses for psychotropic co-medications (in alphabetical order) for patients with vs. without lithium 6](#_Toc188425871)

[Supplementary Table 6. Yearly prescription rates for antidepressants (AD), antipsychotic drugs (AP), tranquilizers (TR), hypnotics (HYP), lithium (Li) and anticonvulsants (AC) in 4,023 patients with depressive episodes (ICD-10 F31.3-5) 7](#_Toc188425872)

[Supplementary Table 7. Yearly prescription rates for antidepressants (AD), antipsychotic drugs (AP), tranquilizers (TR), hypnotics (HYP), lithium (Li) and anticonvulsants (AC) in 3,305 patients with manic episodes (ICD-10 F31.0-2) 8](#_Toc188425873)

[Supplementary Table 8. Yearly rates of lithium (Li) prescriptions in combination with antidepressants (AD), antipsychotic drugs (AP), tranquilizers (TR), hypnotics (HYP), and anticonvulsants (AC) in F31 patients (n = 2796). 9](#_Toc188425874)

[Supplementary Table 9. Yearly prescription rates for with antidepressants (AD), antipsychotic drugs (AP), tranquilizers (TR), hypnotics (HYP), and anticonvulsants (AC) in F31 patients that did not receive lithium (Li) (n = 5911). 10](#_Toc188425875)

[Supplementary Figure 1. Use of lithium and valproic acid in patients in bipolar disorders (F31). Left: only female patients (a,c). Right: only male patient (b, d). Botton: only patient aged 40 or younger (c,d). 11](#_Toc188425876)

[Supplementary Figure 2. Use of psychotropic drugs in patients during a manic episode without psychotic symptoms (F31.1) (a, left) and with psychotic symptoms (F31.2) (b, right). 11](#_Toc188425877)

# Supplementary Table 1. Numbers (%) of prescriptions of second-generation antipsychotics (SGA), mood stabilizers (MS) in total, lithium (Li) and anticonvulsants (AC) for each study year

|  | **SGA** | | **MS** | | **Li** | | **AC** | | **Total-year** |
| --- | --- | --- | --- | --- | --- | --- | --- | --- | --- |
|  | n | % | n | % | n | % | n | % |  |
| 1994 | 6 | 8.33 | 45 | 62.50 | 31 | 43.06 | 20 | 27.78 | 72 |
| 1995 | 6 | 8.96 | 49 | 73.13 | 32 | 47.76 | 25 | 37.31 | 67 |
| 1996 | 11 | 9.65 | 78 | 68.42 | 53 | 46.49 | 36 | 31.58 | 114 |
| 1997 | 17 | 10.30 | 116 | 70.30 | 67 | 40.61 | 71 | 43.03 | 165 |
| 1998 | 28 | 16.47 | 123 | 72.35 | 56 | 32.94 | 77 | 45.29 | 170 |
| 1999 | 43 | 21.29 | 163 | 80.69 | 93 | 46.04 | 92 | 45.54 | 202 |
| 2000 | 59 | 27.83 | 140 | 66.04 | 78 | 36.79 | 81 | 38.21 | 212 |
| 2001 | 101 | 37.69 | 206 | 76.87 | 103 | 38.43 | 138 | 51.49 | 268 |
| 2002 | 115 | 44.23 | 190 | 73.08 | 80 | 30.77 | 136 | 52.31 | 260 |
| 2003 | 163 | 56.79 | 235 | 81.88 | 101 | 35.19 | 167 | 58.19 | 287 |
| 2004 | 251 | 57.31 | 332 | 75.80 | 129 | 29.45 | 249 | 56.85 | 438 |
| 2005 | 314 | 59.81 | 421 | 80.19 | 163 | 31.05 | 316 | 60.19 | 525 |
| 2006 | 351 | 67.76 | 386 | 74.52 | 155 | 29.92 | 278 | 53.67 | 518 |
| 2007 | 255 | 62.81 | 329 | 81.03 | 134 | 33.00 | 233 | 57.39 | 406 |
| 2008 | 309 | 70.55 | 338 | 77.17 | 140 | 31.96 | 248 | 56.62 | 438 |
| 2009 | 326 | 71.65 | 322 | 70.77 | 150 | 32.97 | 220 | 48.35 | 455 |
| 2010 | 355 | 71.29 | 363 | 72.89 | 157 | 31.53 | 258 | 51.81 | 498 |
| 2011 | 391 | 75.19 | 356 | 68.46 | 159 | 30.58 | 231 | 44.42 | 520 |
| 2012 | 398 | 74.81 | 348 | 65.41 | 142 | 26.69 | 244 | 45.86 | 532 |
| 2013 | 379 | 74.02 | 337 | 65.82 | 150 | 29.30 | 218 | 42.58 | 512 |
| 2014 | 406 | 77.19 | 369 | 70.15 | 165 | 31.37 | 239 | 45.44 | 526 |
| 2015 | 367 | 73.84 | 310 | 62.37 | 147 | 29.58 | 196 | 39.44 | 497 |
| 2016 | 385 | 75.79 | 332 | 65.35 | 162 | 31.89 | 203 | 39.96 | 508 |
| 2017 | 422 | 81.62 | 319 | 61.70 | 149 | 28.82 | 210 | 40.62 | 517 |
| **Total** | 5,458 |  | 6207 |  | 2796 |  | 4186 |  | 8707 |

MS: Mood Stabilizers, including exactly lithium, carbamazepine, lamotrigine, and valproic acid.

# Supplementary Table 2. Rates of second-generation antipsychotics (SGA) prescribed; SGAs with less than 25 prescriptions are not shown.

| **SGA** | **n** |
| --- | --- |
| Quetiapine | 2677 |
| Olanzapine | 1536 |
| Risperidone | 832 |
| Aripiprazole | 645 |
| Clozapine | 252 |
| Amisulpride | 164 |
| Ziprasidone | 109 |
| Risperidone microspheres | 62 |
| Clotiapine | 58 |
| Asenapine | 44 |
| Paliperidone | 32 |
| Paliperidone palmitate | 25 |

# Supplement Table 3: Rates (%) of the 10 most common psychotropic drugs in bipolar disorders without and with lithium (a) 1998-2007 (b) 2008-2017

(a)

| **Psychotropic medications**  **1998-2007** | **Without Lithium**  (n=2194) | | **With Lithium**  n=1092) | |
| --- | --- | --- | --- | --- |
|  | **n** | **%** | **n** | **%** |
| Valproic acid* | 916 | 41.75 | 197 | 18.04 |
| Lorazepam | 458 | 20.88 | 192 | 17.58 |
| Olanzapine | 453 | 20.65 | 216 | 19.78 |
| Quetiapine | 335 | 15.27 | 138 | 12.64 |
| Carbamazepine* | 331 | 15.09 | 72 | 6.59 |
| Lamotrigine* | 317 | 14.45 | 85 | 7.78 |
| Mirtazapine | 291 | 13.26 | 111 | 10.16 |
| Venlafaxine | 253 | 11.53 | 109 | 9.98 |
| Diazepam | 232 | 10.57 | 116 | 10.62 |
| Risperidone | 212 | 9.66 | 89 | 8.15 |

* p < 0.001

(b)

| **Psychotropic medications**  **2008-2017** | **Without Lithium**  (n=3482) | | **With Lithium**  (n=1521) | |
| --- | --- | --- | --- | --- |
|  | **n** | **%** | **n** | **%** |
| Quetiapine* | 1626 | 46.70 | 578 | 38.00 |
| Valproic acid* | 1321 | 37.94 | 243 | 15.98 |
| Lorazepam | 778 | 22.34 | 299 | 19.66 |
| Olanzapine | 603 | 17.32 | 259 | 17.03 |
| Lamotrigine* | 538 | 15.45 | 140 | 9.20 |
| Aripiprazole* | 462 | 13.27 | 134 | 8.81 |
| Risperidone | 374 | 10.74 | 149 | 9.80 |
| Venlafaxine | 356 | 10.22 | 174 | 11.44 |
| Diazepam* | 317 | 9.10 | 175 | 11.51 |
| Mirtazapine | 315 | 9.05 | 156 | 10.26 |

* p < 0.001

# Supplementary Table 4. Mean number and standard errors (SE) of medications in bipolar patients treated with (n = 2796) vs. without lithium (n = 5911) per year, including psychotropic and non-psychotropic medications.

|  | **Patients with lithium** | | **Patients without lithium** | |
| --- | --- | --- | --- | --- |
| Year | Mean | SE | Mean | SE |
| 1994 | 3.97 | 0.32 | 2.85 | 0.25 |
| 1995 | 4.00 | 0.39 | 4.03 | 0.33 |
| 1996 | 3.66 | 0.24 | 4.02 | 0.29 |
| 1997 | 4.34 | 0.25 | 3.87 | 0.22 |
| 1998 | 4.05 | 0.25 | 3.81 | 0.19 |
| 1999 | 4.37 | 0.23 | 4.16 | 0.22 |
| 2000 | 4.51 | 0.22 | 4.49 | 0.22 |
| 2001 | 4.44 | 0.20 | 4.36 | 0.17 |
| 2002 | 4.41 | 0.26 | 4.64 | 0.19 |
| 2003 | 4.75 | 0.24 | 4.93 | 0.20 |
| 2004 | 4.73 | 0.20 | 4.75 | 0.15 |
| 2005 | 4.77 | 0.16 | 4.71 | 0.14 |
| 2006 | 4.96 | 0.19 | 4.88 | 0.13 |
| 2007 | 5.97 | 0.23 | 5.09 | 0.16 |
| 2008 | 5.42 | 0.22 | 4.84 | 0.16 |
| 2009 | 5.43 | 0.22 | 5.02 | 0.17 |
| 2010 | 5.31 | 0.20 | 4.77 | 0.16 |
| 2011 | 4.77 | 0.18 | 4.64 | 0.14 |
| 2012 | 5.18 | 0.23 | 4.91 | 0.15 |
| 2013 | 5.11 | 0.22 | 4.78 | 0.15 |
| 2014 | 5.35 | 0.20 | 4.98 | 0.15 |
| 2015 | 5.65 | 0.24 | 4.66 | 0.13 |
| 2016 | 5.17 | 0.20 | 4.95 | 0.15 |
| 2017 | 5.21 | 0.22 | 5.00 | 0.16 |

A paired t-test yields a statistically significant higher number of drugs in patients with lithium (p<0.01), yearly mean with lithium = 4.8; yearly, mean without lithium = 4.5). Only psychotropic drugs: yearly mean with lithium = 3.4; yearly mean without lithium = 2.9 (p<0.01).

# Supplementary Table 5. Median daily doses for psychotropic co-medications (in alphabetical order) for patients with vs. without lithium

|  | **Patients with lithium** | | **Patients without lithium** | |
| --- | --- | --- | --- | --- |
|  | n | Median (mg/day) | n | Median (mg/day) |
| Aripiprazole | 144 | 15 | 501 | 15 |
| Carbamazepine | 133 | 600 | 531 | 600 |
| Lamotrigine | 225 | 175 | 855 | 125 |
| Mirtazapine | 272 | 30 | 609 | 30 |
| Olanzapine | 478 | 15 | 1058 | 15 |
| Quetiapine | 716 | 300 | 1961 | 300 |
| Risperidone | 240 | 3 | 592 | 3 |
| Valproaic acid | 449 | 1300 | 2261 | 1200 |
| Venlafaxine | 288 | 212.5 | 616 | 150 |

# Supplementary Table 6. Yearly prescription rates for antidepressants (AD), antipsychotic drugs (AP), tranquilizers (TR), hypnotics (HYP), lithium (Li) and anticonvulsants (AC) in 4,023 patients with depressive episodes (ICD-10 F31.3-5)

| **Year** | **AD** | | **AP** | | **TR** | | **HYP** | | **Li** | | **AC** | | **Total-year** |
| --- | --- | --- | --- | --- | --- | --- | --- | --- | --- | --- | --- | --- | --- |
|  | n | % | n | % | n | % | n | % | n | % | n | % |  |
| 1994 | 12 | 80.0 | 7 | 46.7 | 5 | 33.3 | 2 | 13.3 | 8 | 53.3 | 4 | 26.7 | 15 |
| 1995 | 25 | 80.6 | 18 | 58.1 | 7 | 22.6 | 6 | 19.4 | 16 | 51.6 | 12 | 38.7 | 31 |
| 1996 | 50 | 90.9 | 19 | 34.5 | 8 | 14.5 | 8 | 14.5 | 24 | 43.6 | 13 | 23.6 | 55 |
| 1997 | 75 | 86.2 | 42 | 48.3 | 26 | 29.9 | 14 | 16.1 | 30 | 34.5 | 31 | 35.6 | 87 |
| 1998 | 63 | 77.8 | 38 | 46.9 | 22 | 27.2 | 14 | 17.3 | 31 | 38.3 | 33 | 40.7 | 81 |
| 1999 | 80 | 82.5 | 35 | 36.1 | 25 | 25.8 | 27 | 27.8 | 43 | 44.3 | 41 | 42.3 | 97 |
| 2000 | 90 | 83.3 | 56 | 51.9 | 32 | 29.6 | 32 | 29.6 | 31 | 28.7 | 38 | 35.2 | 108 |
| 2001 | 95 | 86.4 | 55 | 50.0 | 34 | 30.9 | 23 | 20.9 | 35 | 31.8 | 60 | 54.5 | 110 |
| 2002 | 108 | 85.7 | 67 | 53.2 | 47 | 37.3 | 38 | 30.2 | 37 | 29.4 | 61 | 48.4 | 126 |
| 2003 | 109 | 79.0 | 91 | 65.9 | 58 | 42.0 | 27 | 19.6 | 42 | 30.4 | 92 | 66.7 | 138 |
| 2004 | 169 | 82.0 | 131 | 63.6 | 82 | 39.8 | 56 | 27.2 | 63 | 30.6 | 118 | 57.3 | 206 |
| 2005 | 176 | 78.6 | 141 | 62.9 | 76 | 33.9 | 52 | 23.2 | 70 | 31.3 | 143 | 63.8 | 224 |
| 2006 | 188 | 81.0 | 160 | 69.0 | 88 | 37.9 | 45 | 19.4 | 72 | 31.0 | 114 | 49.1 | 232 |
| 2007 | 151 | 83.9 | 117 | 65.0 | 72 | 40.0 | 44 | 24.4 | 67 | 37.2 | 110 | 61.1 | 180 |
| 2008 | 156 | 82.5 | 123 | 65.1 | 76 | 40.2 | 43 | 22.8 | 67 | 35.4 | 103 | 54.5 | 189 |
| 2009 | 178 | 80.9 | 163 | 74.1 | 94 | 42.7 | 46 | 20.9 | 79 | 35.9 | 109 | 49.5 | 220 |
| 2010 | 166 | 75.1 | 168 | 76.0 | 76 | 34.4 | 34 | 15.4 | 59 | 26.7 | 135 | 61.1 | 221 |
| 2011 | 179 | 74.0 | 184 | 76.0 | 78 | 32.2 | 31 | 12.8 | 68 | 28.1 | 110 | 45.5 | 242 |
| 2012 | 180 | 72.9 | 202 | 81.8 | 76 | 30.8 | 36 | 14.6 | 69 | 27.9 | 116 | 47.0 | 247 |
| 2013 | 174 | 76.0 | 171 | 74.7 | 64 | 27.9 | 23 | 10.0 | 75 | 32.8 | 92 | 40.2 | 229 |
| 2014 | 186 | 75.0 | 199 | 80.2 | 91 | 36.7 | 25 | 10.1 | 87 | 35.1 | 120 | 48.4 | 248 |
| 2015 | 175 | 74.8 | 184 | 78.6 | 72 | 30.8 | 24 | 10.3 | 66 | 28.2 | 100 | 42.7 | 234 |
| 2016 | 194 | 77.3 | 193 | 76.9 | 77 | 30.7 | 32 | 12.7 | 82 | 32.7 | 107 | 42.6 | 251 |
| 2017 | 166 | 65.9 | 202 | 80.2 | 71 | 28.2 | 34 | 13.5 | 73 | 29.0 | 108 | 42.9 | 252 |
| **Total** | 3145 |  | 2766 |  | 1357 |  | 716 |  | 1294 |  | 1970 |  | 4023 |

# Supplementary Table 7. Yearly prescription rates for antidepressants (AD), antipsychotic drugs (AP), tranquilizers (TR), hypnotics (HYP), lithium (Li) and anticonvulsants (AC) in 3,305 patients with manic episodes (ICD-10 F31.0-2)

| **Year** | **AD** | | **AP** | | **TR** | | **HYP** | | **Li** | | **AC** | | **Total-year** |
| --- | --- | --- | --- | --- | --- | --- | --- | --- | --- | --- | --- | --- | --- |
|  | n | % | n | % | n | % | n | % | n | % | n | % |  |
| 1994 | 4 | 19.0 | 17 | 81.0 | 5 | 23.8 | 2 | 9.5 | 13 | 61.9 | 10 | 47.6 | 21 |
| 1995 | 5 | 20.0 | 18 | 72.0 | 4 | 16.0 | 4 | 16.0 | 13 | 52.0 | 10 | 40.0 | 25 |
| 1996 | 11 | 21.6 | 39 | 76.5 | 12 | 23.5 | 1 | 2.0 | 25 | 49.0 | 20 | 39.2 | 51 |
| 1997 | 2 | 3.3 | 52 | 85.2 | 14 | 23.0 | 4 | 6.6 | 31 | 50.8 | 31 | 50.8 | 61 |
| 1998 | 14 | 17.7 | 60 | 75.9 | 22 | 27.8 | 11 | 13.9 | 22 | 27.8 | 38 | 48.1 | 79 |
| 1999 | 23 | 25.6 | 66 | 73.3 | 24 | 26.7 | 19 | 21.1 | 43 | 47.8 | 44 | 48.9 | 90 |
| 2000 | 12 | 15.0 | 62 | 77.5 | 27 | 33.8 | 17 | 21.3 | 39 | 48.8 | 38 | 47.5 | 80 |
| 2001 | 21 | 16.9 | 100 | 80.6 | 45 | 36.3 | 15 | 12.1 | 53 | 42.7 | 76 | 61.3 | 124 |
| 2002 | 13 | 12.3 | 89 | 84.0 | 34 | 32.1 | 15 | 14.2 | 37 | 34.9 | 68 | 64.2 | 106 |
| 2003 | 23 | 21.7 | 88 | 83.0 | 43 | 40.6 | 19 | 17.9 | 42 | 39.6 | 67 | 63.2 | 106 |
| 2004 | 29 | 17.3 | 137 | 81.5 | 60 | 35.7 | 24 | 14.3 | 45 | 26.8 | 113 | 67.3 | 168 |
| 2005 | 36 | 17.9 | 163 | 81.1 | 78 | 38.8 | 28 | 13.9 | 58 | 28.9 | 127 | 63.2 | 201 |
| 2006 | 37 | 19.6 | 163 | 86.2 | 74 | 39.2 | 24 | 12.7 | 57 | 30.2 | 117 | 61.9 | 189 |
| 2007 | 28 | 19.0 | 123 | 83.7 | 59 | 40.1 | 24 | 16.3 | 36 | 24.5 | 99 | 67.3 | 147 |
| 2008 | 28 | 16.4 | 155 | 90.6 | 77 | 45.0 | 20 | 11.7 | 48 | 28.1 | 103 | 60.2 | 171 |
| 2009 | 30 | 17.4 | 147 | 85.5 | 70 | 40.7 | 24 | 14.0 | 54 | 31.4 | 93 | 54.1 | 172 |
| 2010 | 20 | 11.2 | 159 | 89.3 | 76 | 42.7 | 25 | 14.0 | 70 | 39.3 | 97 | 54.5 | 178 |
| 2011 | 31 | 16.4 | 174 | 92.1 | 72 | 38.1 | 19 | 10.1 | 63 | 33.3 | 98 | 51.9 | 189 |
| 2012 | 26 | 13.3 | 176 | 89.8 | 88 | 44.9 | 12 | 6.1 | 57 | 29.1 | 93 | 47.4 | 196 |
| 2013 | 37 | 19.0 | 181 | 92.8 | 69 | 35.4 | 21 | 10.8 | 54 | 27.7 | 101 | 51.8 | 195 |
| 2014 | 34 | 16.2 | 190 | 90.5 | 83 | 39.5 | 14 | 6.7 | 63 | 30.0 | 101 | 48.1 | 210 |
| 2015 | 22 | 12.0 | 161 | 88.0 | 67 | 36.6 | 13 | 7.1 | 51 | 27.9 | 86 | 47.0 | 183 |
| 2016 | 25 | 13.7 | 168 | 91.8 | 77 | 42.1 | 15 | 8.2 | 56 | 30.6 | 78 | 42.6 | 183 |
| 2017 | 24 | 13.3 | 163 | 90.6 | 60 | 33.3 | 14 | 7.8 | 53 | 29.4 | 88 | 48.9 | 180 |
| **Total** | 535 |  | 2851 |  | 1240 |  | 384 |  | 1083 |  | 1796 |  | 3305 |

# Supplementary Table 8. Yearly rates of lithium (Li) prescriptions in combination with antidepressants (AD), antipsychotic drugs (AP), tranquilizers (TR), hypnotics (HYP), and anticonvulsants (AC) in F31 patients (n = 2796).

| **Year** | **AD** | | **AP** | | **TR** | | **HYP** | | **Li** | | **AC** | | **Total-year** |
| --- | --- | --- | --- | --- | --- | --- | --- | --- | --- | --- | --- | --- | --- |
|  | n | % | n | % | n | % | n | % | n | % | n | % |  |
| 1994 | 16 | 51.6 | 19 | 61.3 | 9 | 29.0 | 5 | 16.1 | 31 | 100 | 6 | 19.4 | 31 |
| 1995 | 14 | 43.8 | 23 | 71.9 | 9 | 28.1 | 3 | 9.4 | 32 | 100 | 8 | 25.0 | 32 |
| 1996 | 28 | 52.8 | 25 | 47.2 | 8 | 15.1 | 4 | 7.5 | 53 | 100 | 11 | 20.8 | 53 |
| 1997 | 31 | 46.3 | 45 | 67.2 | 12 | 17.9 | 9 | 13.4 | 67 | 100 | 24 | 35.8 | 67 |
| 1998 | 30 | 53.6 | 32 | 57.1 | 10 | 17.9 | 11 | 19.6 | 56 | 100 | 10 | 17.9 | 56 |
| 1999 | 51 | 54.8 | 56 | 60.2 | 21 | 22.6 | 24 | 25.8 | 93 | 100 | 24 | 25.8 | 93 |
| 2000 | 32 | 41.0 | 51 | 65.4 | 24 | 30.8 | 20 | 25.6 | 78 | 100 | 22 | 28.2 | 78 |
| 2001 | 40 | 38.8 | 70 | 68.0 | 27 | 26.2 | 20 | 19.4 | 103 | 100 | 38 | 36.9 | 103 |
| 2002 | 37 | 46.3 | 46 | 57.5 | 19 | 23.8 | 24 | 30.0 | 80 | 100 | 29 | 36.3 | 80 |
| 2003 | 49 | 48.5 | 70 | 69.3 | 40 | 39.6 | 17 | 16.8 | 101 | 100 | 38 | 37.6 | 101 |
| 2004 | 61 | 47.3 | 86 | 66.7 | 44 | 34.1 | 32 | 24.8 | 129 | 100 | 51 | 39.5 | 129 |
| 2005 | 75 | 46.0 | 108 | 66.3 | 58 | 35.6 | 31 | 19.0 | 163 | 100 | 65 | 39.9 | 163 |
| 2006 | 71 | 45.8 | 122 | 78.7 | 63 | 40.6 | 29 | 18.7 | 155 | 100 | 55 | 35.5 | 155 |
| 2007 | 78 | 58.2 | 97 | 72.4 | 62 | 46.3 | 30 | 22.4 | 134 | 100 | 48 | 35.8 | 134 |
| 2008 | 68 | 48.6 | 112 | 80.0 | 70 | 50.0 | 27 | 19.3 | 140 | 100 | 56 | 40.0 | 140 |
| 2009 | 72 | 48.0 | 113 | 75.3 | 61 | 40.7 | 26 | 17.3 | 150 | 100 | 51 | 34.0 | 150 |
| 2010 | 64 | 40.8 | 114 | 72.6 | 63 | 40.1 | 32 | 20.4 | 157 | 100 | 62 | 39.5 | 157 |
| 2011 | 70 | 44.0 | 128 | 80.5 | 56 | 35.2 | 23 | 14.5 | 159 | 100 | 40 | 25.2 | 159 |
| 2012 | 61 | 43.0 | 116 | 81.7 | 56 | 39.4 | 15 | 10.6 | 142 | 100 | 43 | 30.3 | 142 |
| 2013 | 74 | 49.3 | 121 | 80.7 | 47 | 31.3 | 21 | 14.0 | 150 | 100 | 35 | 23.3 | 150 |
| 2014 | 82 | 49.7 | 133 | 80.6 | 65 | 39.4 | 15 | 9.1 | 165 | 100 | 44 | 26.7 | 165 |
| 2015 | 70 | 47.6 | 115 | 78.2 | 46 | 31.3 | 23 | 15.6 | 147 | 100 | 39 | 26.5 | 147 |
| 2016 | 81 | 50.0 | 126 | 77.8 | 54 | 33.3 | 22 | 13.6 | 162 | 100 | 43 | 26.5 | 162 |
| 2017 | 63 | 42.3 | 118 | 79.2 | 40 | 26.8 | 19 | 12.8 | 149 | 100 | 50 | 33.6 | 149 |
| **Total** | 1318 |  | 2046 |  | 964 |  | 482 |  | 2796 |  | 892 |  | 2796 |

# Supplementary Table 9. Yearly prescription rates for with antidepressants (AD), antipsychotic drugs (AP), tranquilizers (TR), hypnotics (HYP), and anticonvulsants (AC) in F31 patients that did not receive lithium (Li) (n = 5911).

| **Year** | **AD** | | **AP** | | **TR** | | **HYP** | | **Li** | | **AC** | | **Total-year** |
| --- | --- | --- | --- | --- | --- | --- | --- | --- | --- | --- | --- | --- | --- |
|  | n | % | n | % | n | % | n | % | n | % | n | % |  |
| 1994 | 26 | 63.4 | 24 | 58.5 | 5 | 12.2 | 3 | 7.3 | 0 | 0 | 14 | 34.1 | 41 |
| 1995 | 20 | 57.1 | 21 | 60.0 | 10 | 28.6 | 8 | 22.9 | 0 | 0 | 18 | 51.4 | 35 |
| 1996 | 35 | 57.4 | 38 | 62.3 | 12 | 19.7 | 5 | 8.2 | 0 | 0 | 25 | 41.0 | 61 |
| 1997 | 51 | 52.0 | 60 | 61.2 | 30 | 30.6 | 14 | 14.3 | 0 | 0 | 51 | 52.0 | 98 |
| 1998 | 53 | 46.5 | 73 | 64.0 | 40 | 35.1 | 18 | 15.8 | 0 | 0 | 68 | 59.6 | 114 |
| 1999 | 61 | 56.0 | 57 | 52.3 | 30 | 27.5 | 27 | 24.8 | 0 | 0 | 70 | 64.2 | 109 |
| 2000 | 80 | 59.7 | 81 | 60.4 | 48 | 35.8 | 34 | 25.4 | 0 | 0 | 66 | 49.3 | 134 |
| 2001 | 94 | 57.0 | 102 | 61.8 | 62 | 37.6 | 27 | 16.4 | 0 | 0 | 111 | 67.3 | 165 |
| 2002 | 98 | 54.4 | 130 | 72.2 | 73 | 40.6 | 34 | 18.9 | 0 | 0 | 117 | 65.0 | 180 |
| 2003 | 106 | 57.0 | 143 | 76.9 | 80 | 43.0 | 39 | 21.0 | 0 | 0 | 145 | 78.0 | 186 |
| 2004 | 165 | 53.4 | 227 | 73.5 | 117 | 37.9 | 60 | 19.4 | 0 | 0 | 221 | 71.5 | 309 |
| 2005 | 176 | 48.6 | 275 | 76.0 | 129 | 35.6 | 61 | 16.9 | 0 | 0 | 270 | 74.6 | 362 |
| 2006 | 187 | 51.5 | 283 | 78.0 | 133 | 36.6 | 61 | 16.8 | 0 | 0 | 242 | 66.7 | 363 |
| 2007 | 140 | 51.5 | 210 | 77.2 | 104 | 38.2 | 53 | 19.5 | 0 | 0 | 203 | 74.6 | 272 |
| 2008 | 150 | 50.3 | 233 | 78.2 | 120 | 40.3 | 49 | 16.4 | 0 | 0 | 208 | 69.8 | 298 |
| 2009 | 158 | 51.8 | 254 | 83.3 | 126 | 41.3 | 57 | 18.7 | 0 | 0 | 189 | 62.0 | 305 |
| 2010 | 164 | 48.1 | 292 | 85.6 | 127 | 37.2 | 36 | 10.6 | 0 | 0 | 223 | 65.4 | 341 |
| 2011 | 178 | 49.3 | 308 | 85.3 | 126 | 34.9 | 36 | 10.0 | 0 | 0 | 210 | 58.2 | 361 |
| 2012 | 167 | 42.8 | 336 | 86.2 | 135 | 34.6 | 45 | 11.5 | 0 | 0 | 220 | 56.4 | 390 |
| 2013 | 163 | 45.0 | 308 | 85.1 | 114 | 31.5 | 34 | 9.4 | 0 | 0 | 203 | 56.1 | 362 |
| 2014 | 167 | 46.3 | 313 | 86.7 | 138 | 38.2 | 29 | 8.0 | 0 | 0 | 218 | 60.4 | 361 |
| 2015 | 155 | 44.3 | 298 | 85.1 | 120 | 34.3 | 25 | 7.1 | 0 | 0 | 185 | 52.9 | 350 |
| 2016 | 170 | 49.1 | 300 | 86.7 | 125 | 36.1 | 27 | 7.8 | 0 | 0 | 184 | 53.2 | 346 |
| 2017 | 156 | 42.4 | 324 | 88.0 | 121 | 32.9 | 37 | 10.1 | 0 | 0 | 187 | 50.8 | 368 |
| **Total** | 2920 |  | 4690 |  | 2125 |  | 819 |  | 0 |  | 3648 |  | 5911 |

# Supplementary Figure 1. Use of lithium and valproic acid in patients in bipolar disorders (F31). Left: only female patients (a,c). Right: only male patient (b, d). Botton: only patient aged 40 or younger (c,d).

Li: Lithium. Val: Valproic Acid


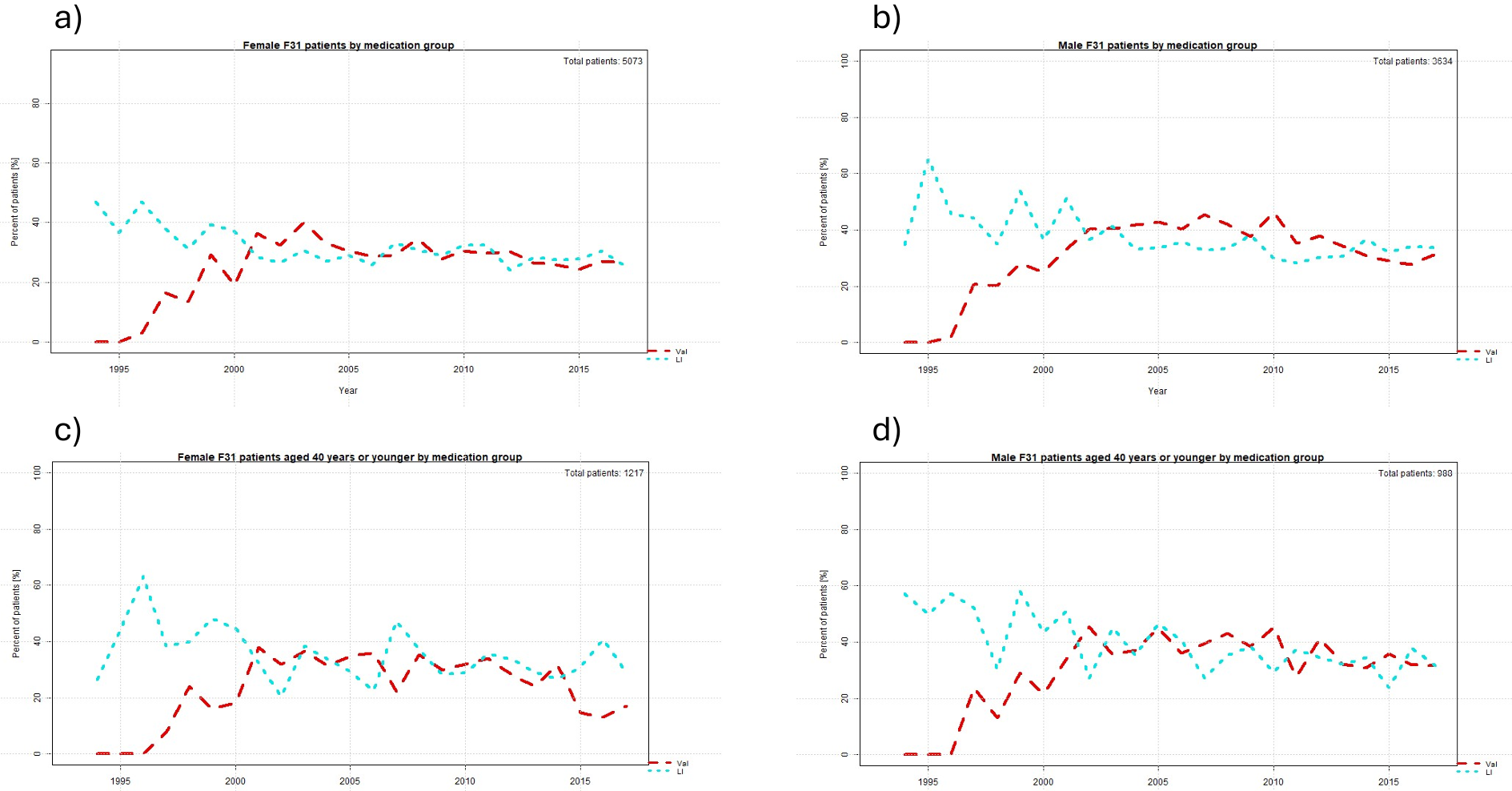


# Supplementary Figure 2. Use of psychotropic drugs in patients during a manic episode without psychotic symptoms (F31.1) (a, left) and with psychotic symptoms (F31.2) (b, right).

AD: Antidepressant drugs. AP: Antipsychotic drugs. TR: Tranquilizers. HYP: Hypnotics. Li: Lithium. AC: Anticonvulsant drugs.


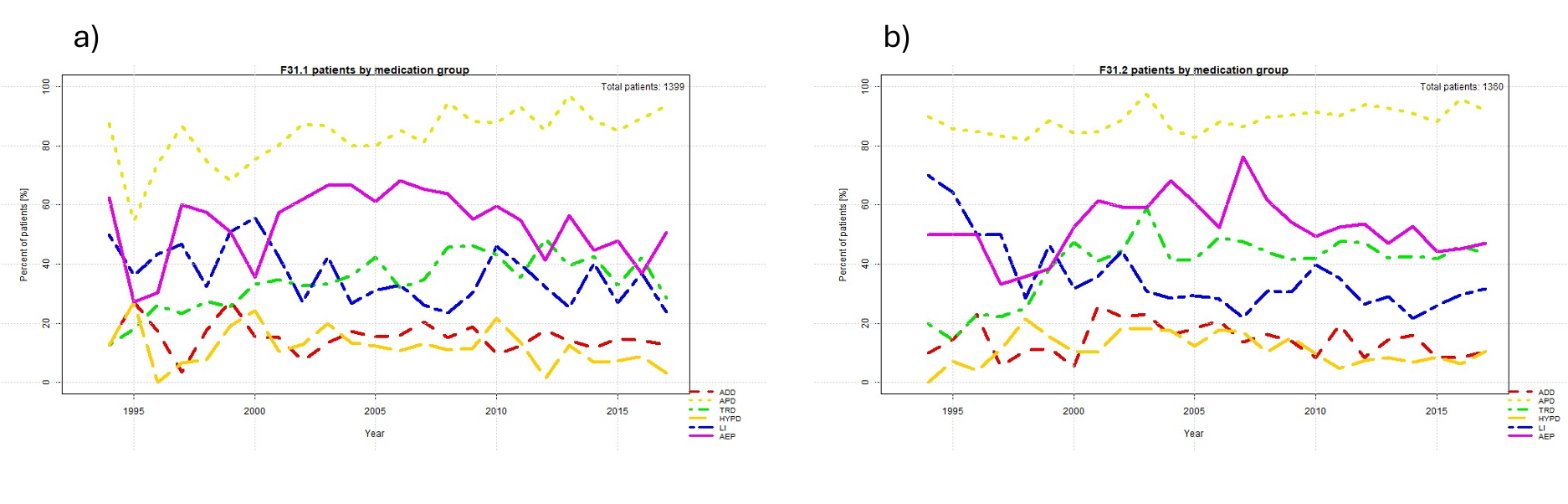

Supplement: Supplementary file 1 — Supplementary Material 1 [file 40345_2025_370_MOESM1_ESM.docx]
